# Heteroepitaxial Growth of High-Quality and Crack-Free AlN Film on Sapphire Substrate with Nanometer-Scale-Thick AlN Nucleation Layer for AlGaN-Based Deep Ultraviolet Light-Emitting Diodes

**DOI:** 10.3390/nano9111634

**Published:** 2019-11-17

**Authors:** Jie Zhao, Hongpo Hu, Yu Lei, Hui Wan, Liyan Gong, Shengjun Zhou

**Affiliations:** 1 Center for Photonics and Semiconductors, School of Power and Mechanical Engineering, Wuhan University, Wuhan 430072, China; zjie1994@whu.edu.cn (J.Z.); huhongpo@whu.edu.cn (H.H.); ly2019202080018@whu.edu.cn (Y.L.); 2019202080016@whu.edu.cn (L.G.); 2 HC SemiTek Corporation, Suzhou 215600, China; 3 The Institute of Technological Sciences, Wuhan University, Wuhan 430072, China; wanhui_hb@whu.edu.cn

**Keywords:** DUV LED, nucleation layer, tensile stress, surface morphology, crystal quality

## Abstract

High-quality and crack-free aluminum nitride (AlN) film on sapphire substrate is the foundation for high-efficiency aluminum gallium nitride (AlGaN)-based deep ultraviolet light-emitting diodes (DUV LEDs). We reported the growth of high-quality and crack-free AlN film on sapphire substrate with a nanometer-scale-thick AlN nucleation layer (NL). Three kinds of nanometer-scale-thick AlN NLs, including in situ low-temperature AlN (LT-AlN) NL, oxygen-undoped ex situ sputtered AlN NL, and oxygen-doped ex situ sputtered AlN NL, were prepared for epitaxial growth of AlN films on sapphire substrates. The influence of nanoscale AlN NL thickness on the optical transmittance, strain state, surface morphology, and threading dislocation (TD) density of the grown AlN film on sapphire substrate were carefully investigated. The average optical transmittance of AlN film on sapphire substrate with oxygen-doped sputtered AlN NL was higher than that of AlN films on sapphire substrates with LT-AlN NL and oxygen-undoped sputtered AlN NL in the 200–270 nm wavelength region. However, the AlN film on sapphire substrate with oxygen-undoped sputtered AlN NL had the lowest TD density among AlN films on sapphire substrates. The AlN film on sapphire substrate with the optimum thickness of sputtered AlN NL showed weak tensile stress, a crack-free surface, and low TD density. Furthermore, a 270-nm AlGaN-based DUV LED was grown on the high-quality and crack-free AlN film. We believe that our results offer a promising and practical route for obtaining high-quality and crack-free AlN film for DUV LED.

## 1. Introduction

Aluminum gallium nitride (AlGaN)-based deep ultraviolet light-emitting diodes (DUV LEDs) have numerous applications, including sterilization, polymer curing, secure communication, and biochemical inspection [1,2,3,4,5]. For AlGaN-based DUV LEDs, aluminum nitride (AlN) film is usually used as the template for the epitaxial growth of the AlGaN layer due to its similar in-plane lattice constant and thermal expansion coefficient [6,7,8,9]. The quality of the AlN film is essential for the epitaxial growth of high-quality AlGaN and high-efficiency DUV LEDs. These AlN films are generally grown on sapphire substrates because sapphire substrates are low-cost and have high DUV-transparency [10,11,12,13,14,15,16]. However, the mismatches of lattice constants and thermal expansion coefficients between AlN film and sapphire substrate induces stress and dislocations, thereby reducing the internal quantum efficiency and degrading the optical and electrical performance of DUV LEDs [17,18,19].

To overcome the problems caused by large lattice and thermal mismatches between AlN and sapphire substrate, the in situ low-temperature AlN (LT-AlN) nucleation layer (NL) has been used as initial growth step before the epitaxy of AlN film on sapphire substrate. The characteristics of AlN film are closely related to the growth condition of AlN NL, which modifies the growth-mode of the subsequent AlN film [20,21,22]. The ex situ nanometer-scale-thick sputtered AlN NL has also been widely applied for the growth of high-quality AlN film on sapphire substrate [23,24,25,26]. However, the sputtering chamber may have contaminants such as oxygen, which can be incorporated into the AlN NL during the sputtering process, impacting the physical properties of sputtered AlN NL [27,28,29]. Understanding the effect of oxygen-doped sputtered AlN NL on the subsequent AlN film is important to the realization of high-efficiency DUV LEDs. On the other hand, the crystalline structure of AlN film is closely related with the thickness of sputtered AlN NL [30,31,32,33,34]. The characteristics of AlN film on sapphire substrate with different thicknesses of sputtered AlN NL is of significant interest for DUV LEDs.

In this study, nanometer-scale-thick LT-AlN NL, oxygen-undoped sputtered AlN NL, and oxygen-doped sputtered AlN NL were prepared for the epitaxial growth of AlN films on sapphire substrates. The influence of nanoscale AlN NL thickness on the optical transmittance, strain state, surface morphology, and threading dislocation (TD) density of AlN film on sapphire substrate was investigated in detail. The optical transmittance, strain state, surface morphology, and crystal quality of AlN films were investigated by UV/Vis/NIR spectrometer, Raman spectroscopy, optical microscopy, atomic force microscopy (AFM) and high-resolution X-ray diffractometer (XRD). By optimizing AlN NL, we successfully grew a high-quality and crack-free AlN film on sapphire substrate and fabricated a 270-nm DUV LED.

## 2. Materials and Methods

### 2.1. Growth of AlN Films on Sapphire Substrates

Prior to the growth of LT-AlN NL, the sapphire substrate was thermally cleaned in H_2_ ambient at 1100 °C for 15 min. Trimethylaluminum (TMAl) and ammonia (NH_3_) were used as precursors for aluminum and nitrogen, respectively. The 25-nm-thick LT-AlN NL was grown on sapphire substrate using an AIXTRON metal-organic chemical vapor deposition (MOCVD) system with a close-coupled showerhead reactor at 1000 °C and 50 mbar under the conditions of H_2_, NH_3_, and TMAl flow rates of 6 slm, 3 slm, and 22 μmol/min, respectively. Nanometer-scale-thick sputtered AlN NL was deposited on *c*-plane sapphire substrate using the NAURA iTops A330 sputter system. An aluminum disk (99.999%) was used as sputtering target. For the deposition of oxygen-doped sputtered AlN NL, the flow rates of N_2_, Ar, and O_2_ were 180 sccm, 60 sccm, and 2 sccm, respectively. The O_2_ was not introduced in the sputtering chamber during the deposition of oxygen-undoped sputtered AlN NL. The sputtering conditions were given as follows: An RF power of 700 W, chamber pressure of 6.7 × 10^−3^ mbar, and heater temperature of 650 °C. The thickness of sputtered AlN NL was controlled by changing the sputtering duration. The epitaxy of AlN film was carried out in an AIXTRON MOCVD system using the same growth condition. The AlN film with thicknesses of approximately 2 μm were grown at 1230 °C under chamber pressure of 50 mbar with V/III ratio of 500.

### 2.2. MOCVD Growth of DUV LED

Trimethylaluminum (TMAl), trimethylgallium (TMGa), and ammonia (NH_3_) were used as Al, Ga, and N precursors, respectively. Bis(cyclopentadienyl)magnesium (Cp_2_Mg) and silane (SiH_4_) were used as p- and n-type dopants. The AlGaN-based DUV LED was grown on AlN film on sapphire substrate with 25-nm-thick oxygen-undoped sputtered AlN NL by MOCVD, including a 15-period AlN (8 nm)/Al_0.85_Ga_0.15_N (12 nm) superlattices (SL) at 1160 °C, a 20-period AlN (4 nm)/Al_0.83_Ga_0.17_N (6 nm) SL at 1160 °C, a 290-nm-thick Al_0.83_Ga_0.17_N layer at 1160 °C, a 200-nm-thick Al_0.65_Ga_0.35_N layer at 1140 °C, and a 1.4-μm-thick heavily doped n^+^-Al_0.65_Ga_0.35_N layer (Si doping = 1.5 × 10^19^ cm^−3^) at 1140 °C, a six-period Al_0.35_Ga_0.65_N (1.5 nm)/Al_0.55_Ga_0.45_N (12 nm) multiple quantum wells (MQWs) at 1000 °C, a 6-period p-Al_0.8_Ga_0.2_N (3 nm)/Al_0.6_Ga_0.4_N (7 nm) SL, and a 80-nm-thick p-GaN layer (Mg doping = 1 × 10^20^ cm^−3^) at 1000 °C. After the epitaxial growth, the DUV LED wafer was annealed at 800 °C with N_2_ flow to activate the Mg acceptors.

### 2.3. Characterization

The cross-sectional structure of AlN film on sapphire substrate was characterized by field emission scanning electron microscope (SEM, Oxford Instruments, London, UK) under an acceleration voltage of 5.0 kV. The optical transmittance of AlN film on sapphire substrate was measured using an UV/Vis/NIR spectrometer (PerkinElmer, Waltham, Massachusetts, USA). The glass microscope slide was used as reference sample for optical transmittance characterization. The AlN film on sapphire substrate with 25-nm-thick oxygen-doped sputtered AlN NL was selected for secondary ion mass spectroscopy (SIMS, Cameca, Paris, France) analysis. The stress state of AlN film on sapphire substrates was evaluated by means of Raman spectroscopy (Renishaw, London, UK). Raman measurement was performed using confocal Raman microscope (Renishaw, London, UK) at room temperature. A He-Ne laser with a 633-nm line was used for excitation and was focused on sample surface to a spot size of 1 μm by an objective lens with magnification of 100 times and high numerical aperture of 0.85. The Raman spectrum was recorded in back scattering geometry with laser beam incident on the (0001) surface. The surface morphology of AlN film on sapphire substrate was taken by Nomarski differential interference contrast (DIC) microscopy (Leica, Wetzlar, Germany). The optical DIC microscopy image of AlN film on sapphire substrate was conducted at the magnification of 1000 times. The crystal quality of AlN films on sapphire substrates was characterized by high-resolution XRD (BEDE, Durham, UK) with Cu Kα1 radiation source (λ = 0.15405 nm) and Ge(004) monochromator operated at 40 kV and 40 mA. The AFM (JPK, Berlin, Germany), performed on NanoWizard 4 in tapping mode, was used to determine the etch pit densities of AlN film. Cross-sectional transmission electron microscopy (TEM) images of DUV LED epitaxial structure were taken with a FEI Tecnai F20 system (FEI, Hillsboro, OR, USA) at 200 kV, and the TEM sample was prepared by focus ion beam milling using Ga ions at 30 kV. The photoluminescence (PL) measurement were carried out by exciting the Al_0.35_Ga_0.65_N/Al_0.55_Ga_0.45_N MQWs with a 248 nm KrF laser at room temperature. A semiconductor parameter analyzer (Keysight, Telford, UK) was used to measure current-voltage (I-V) characteristic of DUV LED.

### 2.4. Device Fabrication

For fabrication of the DUV LED chip, a mesa depth of about 0.9 μm was etched to expose the n-Al_0.65_Ga_0.35_N layer using inductively coupled plasma (ICP) etching system (SPTS, Palo Alto, CA, USA) based on BCl_3_/Cl_2_ mixture gas [35]. Ti/Al metal stack was deposited on the n-Al_0.65_Ga_0.35_N layer, which was then annealed in N_2_ for 1 min at 900 °C. The Ni/Au current spreading layer was deposited on p-GaN and then annealed in O_2_ for 5 min at 550 °C. Finally, the DUV LED wafer was thinned to approximately 150 μm and diced into chips with size of 950 µm × 550 µm.

## 3. Results and Discussion

**Figure 1:**
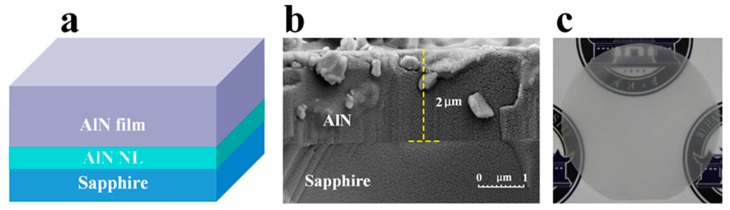
(**a**) Schematic illustration of aluminum nitride (AlN) film on sapphire substrate. (**b**) Cross-sectional scanning electron microscopy (SEM) image of AlN film on sapphire substrate with an AlN nucleation layer (NL). (**c**) Photograph of the 2-in. AlN film on sapphire substrate showing a crack-free surface.

Figure 1a depicts schematic of AlN film on sapphire substrate with AlN NL. The cross-sectional SEM image in Figure 1b exhibited a very sharp heterointerface between AlN and sapphire substrate. The thickness of AlN epilayer was measured to be 2.0 μm. Figure 1c shows the photograph of 2-in. AlN film on sapphire substrate. The AlN film on sapphire substrate had a smooth and crack-free surface, which benefitted the performance of DUV LED.

**Figure 2:**
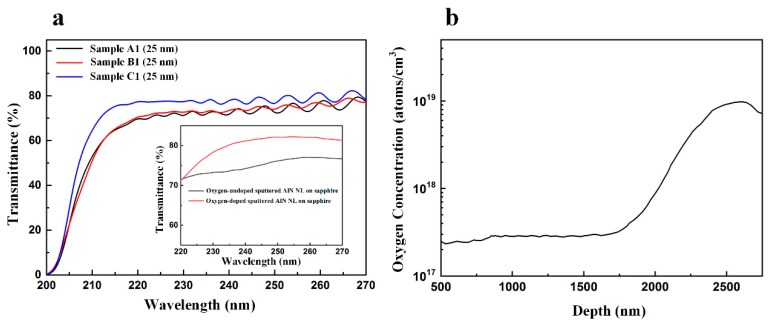
(**a**) Optical transmittance spectra of Sample A1, Sample B1, and Sample C1. (**b**) SIMS depth profiles of oxygen in Sample C1.

We analyzed the optical property of AlN films on sapphire substrates with LT-AlN NL, oxygen-undoped sputtered AlN NL, and oxygen-doped sputtered AlN NL using an UV/Vis/NIR spectrometer. The AlN films on sapphire substrates with 25-nm-thick LT-AlN NL, 25-nm-thick oxygen-undoped sputtered AlN NL, and 25-nm-thick oxygen-doped sputtered AlN NL were labelled as Sample A1, Sample B1, and Sample C1, respectively. Figure 2a shows the optical transmittance spectra of Sample A1–C1 in the 200–270 nm wavelength region. The average optical transmittance of Sample A1–C1 were 64.3%, 64.7%, and 69.8%, respectively. The average optical transmittance of Sample C1 was about 8% higher than that of Sample A1 and Sample B1. As shown in the inset of Figure 2a, the average optical transmittance of oxygen-doped sputtered AlN NL on sapphire was about 7% higher than that of oxygen-undoped sputtered AlN NL on sapphire in the wavelength region (λ = 220–270 nm). Based on the comparison of optical transmittance between the AlN film and sputtered AlN NL, we assumed that the AlN NL was directly responsible for the variation of the optical transmittance. The incorporation of oxygen into the AlN layer would affect the optical properties of the AlN film on sapphire substrate [27,28,29]. The incorporation of oxygen into the growing AlN layer was inevitable during the high-temperature MOCVD growth process [36,37]. Carryover of oxygen into the 2-µm AlN film from the oxygen-doped sputtered AlN NL was also possible during the growth process. Compared with oxygen-undoped sputtered AlN NL, oxygen-doped sputtered AlN NL had a greater probability of carrying over of oxygen into the 2-µm AlN film. As a result, the optical transmittance of Sample C1 was higher than that of Sample B1 in wavelengths ranging from 200 nm to 270 nm. However, the underlying physical mechanism which caused the oxygen dopant to increase the optical transmittance of AlN film in the DUV wavelength range is unclear and will need further investigation. Figure 2b shows the SIMS depth profiles of oxygen in Sample C1. The measured concentration of oxygen atoms in the oxygen-doped sputtered AlN NL and 2-um-thick AlN film was about 1 × 10^19^ cm^−3^ and 2.5 × 10^17^ cm^−3^, respectively.

**Figure 3:**
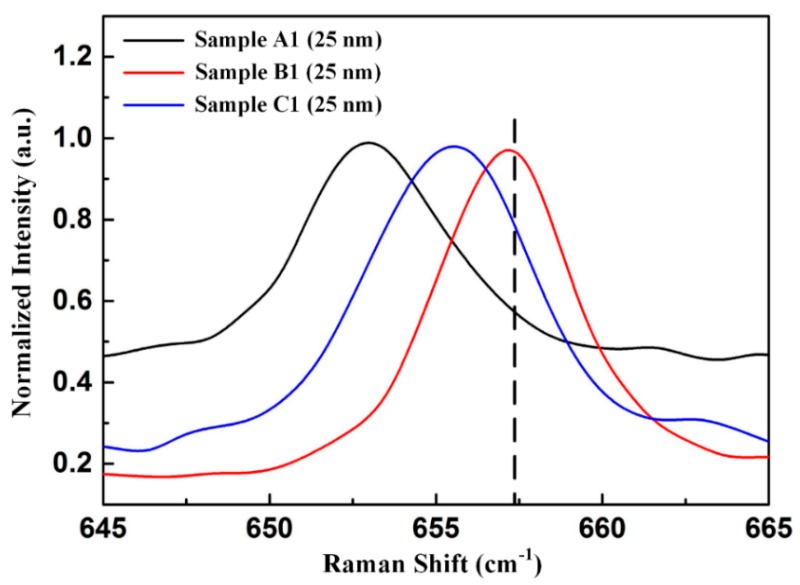
Normalized Raman spectra of E_2_ (high) mode for Sample A1, Sample B1, and Sample C1.

We used Raman spectroscopy to ascertain the stress states of Sample A1–C1. Figure 3 shows normalized Raman spectra of E_2_ (high) mode for these samples. The vertical dotted line shows the E_2_ (high) peak position for the stress-free bulk AlN with a wavenumber of 657.4 cm^−1^. Raman peaks of E_2_ (high) mode in Sample A1–C1 were located at 653.4 cm^−1^, 657.1 cm^−1^, and 655.2 cm^−1^, respectively. The Raman frequency shifted to the higher-frequency side of the stress-free position (blue shifts) due to the existence of residual compressive stress and to the lower-frequency side (red shifts) due to the existence of residual tensile stress [38]. Compared with the Raman peak of E_2_ (high) mode in the stress-free bulk AlN located at 657.4 cm^−1^, these samples exhibited red shifts of Raman peaks, indicating the existence of tensile stresses in the AlN films. It has been reported that the stress in crystal has a linear relationship with frequency shift [39]. In the linear approximation, the stress can be given by

(1) $$\sigma =\frac{∆\omega }{k},$$

where ∆*ω* is the shift of frequency of phonon, *k* is the Raman stress coefficient (3 cm^−1^/GPa) for E_2_ (high) mode of AlN, and σ is the stress. From Equation (1), the tensile stresses of Sample A1–C1 were determined to be 1333 MPa, 100 MPa, and 733 MPa, respectively.

**Figure 4:**
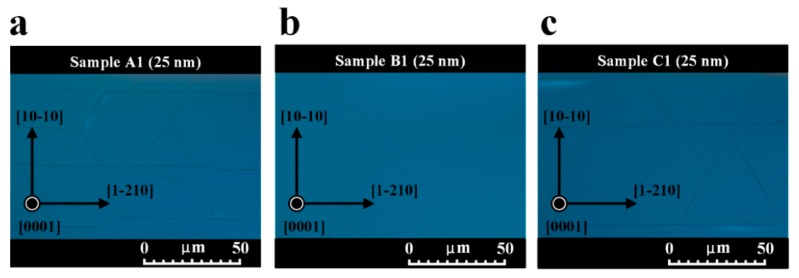
Plan-view optical differential interference contrast (DIC) images of (**a**) Sample A1, (**b**) Sample B1, and (**c**) Sample C1 conducted at the magnification of 1000 times.

We examined the role of stress states in affecting the surface morphologies of Sample A1–C1 by DIC microscopy. Figure 4a–c show the plan-view optical DIC images of Sample A1–C1, respectively. Cracks in Sample A1, as shown in Figure 4a, were caused by excessive tensile stress. The cracks were found along the [1-210] directions, which corresponded to the {10-10} cleavage planes. Due to lower surface energy for the {10-10} planes as compared to the {1-210} planes, the strain energy can be released in the form of crack along {10-10} planes in the AlN film on sapphire substrate [40]. As depicted in Figure 4b, the smooth and crack-free surface of Sample B1 was obtained, revealing that tensile strain was effectively relaxed in the AlN film on sapphire substrate. Cracks were also generated in the surface of Sample C1 along the [1-210] directions, as illustrated in Figure 4c. The plan-view DIC images of AlN films were consistent with the results obtained from calculated tensile stresses of AlN films in Figure 3.

**Figure 5:**
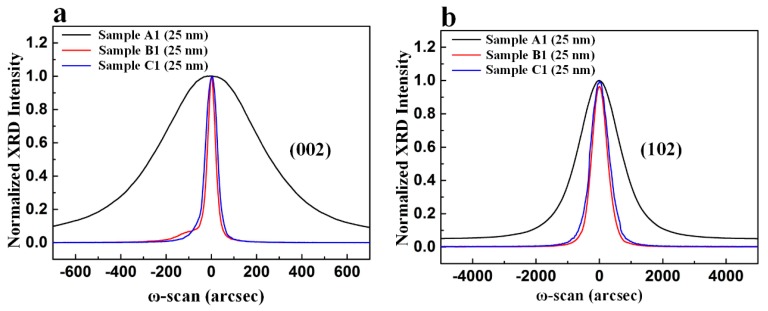
(**a**) Symmetric (002) and (**b**) asymmetric (102) ω-scan rocking curves of Sample A1, Sample B1, and Sample C1.

We studied the TD densities of Sample A1–C1 using high-resolution XRD. It was previously reported that the full width at half maximum (FWHMs) of symmetric (002) and asymmetric (102) rocking curves are mainly influenced by screw and edge dislocation density, respectively [41,42]. Generally, the smaller FWHM values of rocking curves, the lower TD densities of sample. Therefore, we adopted the measured FWHM values of symmetric (002) and asymmetric (102) rocking curves to compare the TD densities of these samples. Figure 5a,b depict symmetric (002) and asymmetric (102) ω-scan rocking curves of these samples. As shown in Figure 5a, the FWHMs of symmetric (002) reflection plane for Sample A1–C1 were measured to be 497 arcsec, 45 arcsec, and 60 arcsec, respectively. As shown in Figure 5b, the FWHMs of asymmetric (102) reflection plane for Sample A1–C1 were measured to be 1641 arcsec, 552 arcsec, and 695 arcsec, respectively. Compared with LT-AlN NL, sputtered AlN NL was composed of smaller and more uniform grains with better c-axis orientation, leading to better growth-mode modification in the subsequent growth process [43,44]. Therefore, both screw and edge dislocation densities of Sample B1 and Sample C1 were lower than those of Sample A1. Since oxygen may form complexes with Al, Al atoms involved in the formation of these complexes distorted the Al sublattice [45,46]. As a result, the TD density of Sample B1 was lower than that of Sample C1.

**Figure 6:**
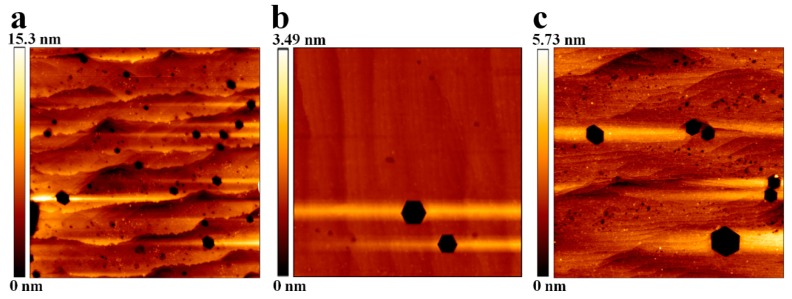
Atomic force microscopy (AFM) images (5 × 5 μm^2^) of etching pits in (**a**) Sample A1, (**b**) Sample B1, and (**c**) Sample C1 after molten KOH etching at 185 °C for 10 min.

We obtained the TD density of Sample A1, Sample B1, and Sample C1 by counting the number of etch pits in the 5 × 5 µm^2^ AFM images. The etch pits on the top surface of these samples were formed by chemical etching in a molten KOH at 185 °C for 10 min. It has been reported that TDs are associated with etch pits [44]. Figure 6a–c shows the 5 × 5 μm^2^ AFM images of etch pits in Sample A1–C1. From the AFM images of Figure 6a–c, the etch pit densities of Sample A1–C1 were calculated to be 7.4 × 10^8^ cm^−2^, 4.8 × 10^7^ cm^−2^, and 5.9 × 10^8^ cm^−2^, respectively. Owing to non-uniform grains, the TD density of Sample A1 was higher than that of Sample B1 and Sample C1. The TD density from AFM measurement was in good agreement with the results of XRD analyses.

**Figure 7:**
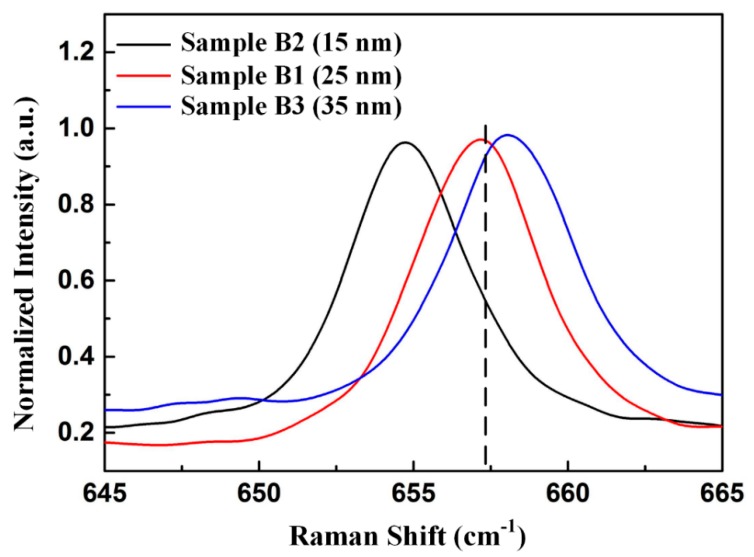
Normalized Raman spectra of E_2_ (high) mode for Sample B1, Sample B2, and Sample B3.

We explored the effect of thickness of oxygen-undoped sputtered AlN NL on the stress in AlN film on sapphire substrate by Raman spectroscopy. The AlN film on sapphire substrate with 15-nm-thick and 35-nm-thick oxygen-undoped sputtered AlN NL were labelled as Sample B2 and Sample B3, respectively. Figure 7 shows normalized Raman spectra of the E_2_ (high) mode for Sample B1–B3. Raman peaks of E_2_ (high) mode in Sample B2, Sample B1, and Sample B3 located at 654.8 cm^−1^, 657.1 cm^−1^, and 658.2 cm^−1^, respectively. Sample B2 exhibited red shifts of Raman peak, indicating the existence of tensile stress in Sample B2. The tensile stress of Sample B2 was calculated to be 867 MPa. However, Sample B3 exhibited blue shifts of Raman peak, indicating the existence of compressive stress in Sample B3. The compressive stress of Sample B3 was calculated to be 267 MPa.

**Figure 8:**
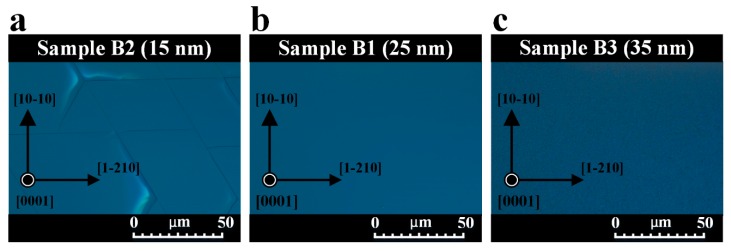
Plan-view optical DIC images of (**a**) Sample B2, (**b**) Sample B1, and (**c**) Sample B3 conducted at the magnification of 1000 times.

We analyzed the effect of residual stress on the surface morphologies of Sample B1, Sample B2, and Sample B3 using optical microscopy. Figure 8 shows optical microscopic surface morphologies of these three samples. A crack network, which was caused by excessive tensile stress, can be observed in Sample B2 shown in Figure 8a. Sample B1 exhibited a smooth surface, as shown in Figure 8b. As illustrated in Figure 8c, the surface of Sample B3 was covered with hillocks. Compared with 25-nm-thick oxygen-undoped sputtered AlN NL, the decrease or increase in thickness of oxygen-undoped sputtered AlN NL had a negative effect on the surface morphology of AlN film.

**Figure 9:**
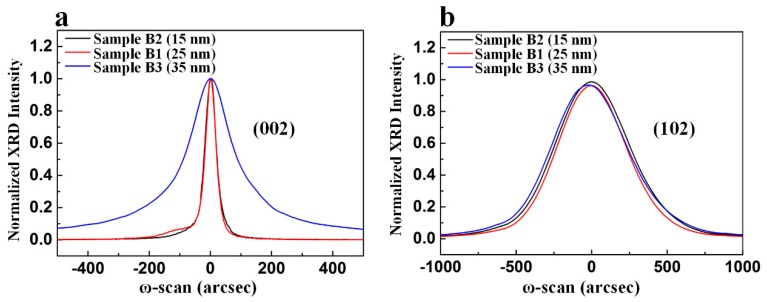
(**a**) Symmetric (002) and (**b**) asymmetric (102) ω-scan rocking curves with normalized peak intensity for Sample B1, Sample B2, and Sample B3.

We investigated the influence of nanoscale oxygen-undoped sputtered AlN NL thickness on the crystal quality of AlN film on sapphire substrate by XRD. Figure 9a,b show symmetric (002) and asymmetric (102) ω-scan rocking curves with normalized peak intensity for these three samples, respectively. The FWHMs of the symmetric (002) reflection plane for Sample B2, Sample B1, and Sample B3 were 47 arcsec, 45 arcsec, and 207 arcsec, respectively, as shown in Figure 9a. The FWHMs of the asymmetric (102) reflection plane for Sample B2, Sample B1, and Sample B3 were 593 arcsec, 552 arcsec, and 613 arcsec, respectively, as shown in Figure 9b. Both the screw and edge dislocation densities of Sample B1 were lower than those of Sample B2 and Sample B3.

**Figure 10:**
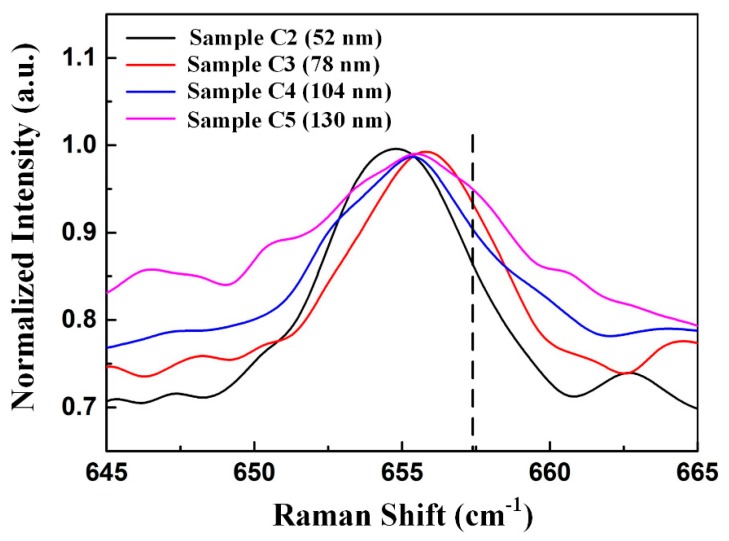
Normalized Raman spectra of the E_2_ (high) mode for Sample C2, Sample C3, Sample C4, and Sample C5.

We studied the effect of nanoscale oxygen-doped sputtered AlN NL thickness on the residual stress of AlN film on sapphire substrate using Raman spectroscopy. The AlN film on sapphire substrate with 52-nm-thick, 78-nm-thick, 104-nm-thick, and 130-nm-thick oxygen-doped sputtered AlN NL were labelled as Sample C2, Sample C3, Sample C4, and Sample C5, respectively. Figure 10 shows normalized Raman spectra of the E_2_ (high) mode for these samples. The Raman peaks of E_2_ (high) mode in Sample C1–C5 located at 655.2 cm^−1^, 654.8 cm^−1^, 655.7 cm^−1^, 655.2 cm^−1^, and 655.1 cm^−1^, respectively. The tensile stresses of Sample C1–C5 were calculated to be 733 MPa, 867 MPa, 567 MPa, 733 MPa, and 767 MPa. Sample C3 had the weakest tensile stress.

**Figure 11:**
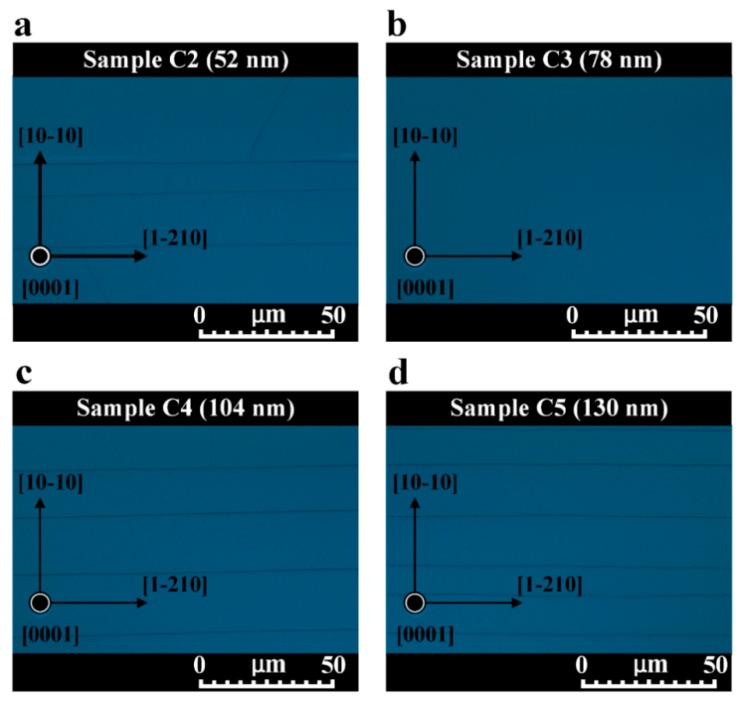
Plan-view optical DIC images of (**a**) Sample C2, (**b**) Sample C3, (**c**) Sample C4, and (**d**) Sample C5 conducted at the magnification of 1000 times.

We analyzed the influence of residual stress on the surface morphologies of Sample C2, Sample C3, Sample C4, and Sample C5 using optical microscopy. Figure 11a–d show surface morphologies of these samples. The density of cracks in the surface of AlN film decreased as the thickness of oxygen-doped sputtered AlN NL was increased from 25 nm to 78 nm but increased as the thickness of oxygen-doped sputtered AlN NL was increased from 78 nm to 130 nm. Among these samples, Sample C3 had a crack-free surface. The trend of changes in density of cracks was consistent with the variation of calculated tensile stresses shown in Figure 7.

**Figure 12:**
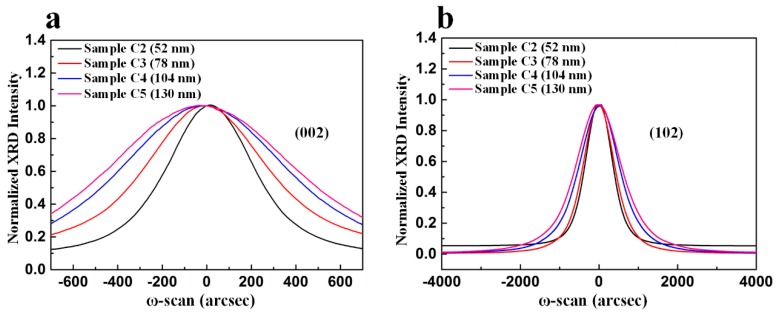
(**a**) Symmetric (002) and (**b**) asymmetric (102) ω-scan rocking curves with normalized peak intensity for Sample C2, Sample C3, Sample C4, and Sample C5.

We investigated the effect of nanoscale oxygen-doped sputtered AlN NL thickness on crystal quality of these samples by XRD. Figure 12a,b illustrate symmetric (002) and asymmetric (102) ω-scan rocking curves of these four samples. The FWHMs of the symmetric (002) reflection plane for Samples C1–C5 were 60, 435, 568, 757, and 914 arcsec, respectively, as illustrated in Figure 12a. The FWHMs of the asymmetric (102) reflection plane for Samples C1–C5 were 695, 713, 911, 1130, and 1362 arcsec, respectively, as shown in Figure 12b. Sample C1 had the lowest TD density.

Based on the results mentioned above, a high-quality and crack-free AlN film on sapphire substrate was obtained by employing 25-nm-thick oxygen-undoped sputtered AlN NL. Uesugi et al. reported that the FWHMs of the symmetric (002) reflection planes for the AlN films were lower than 12 arcsec before and after high-temperature annealing, and that the FWHMs of the symmetric (102) reflection planes for the AlN films were about 3000 arcsec and 150 arcsec before and after high-temperature annealing [41,47,48,49]. The FWHM of AlN (102) X-ray rocking curves for our sample is lower when compared to the previously reported result before high-temperature annealing. However, the FWHM of AlN (102) X-ray rocking curves for our sample is higher when compared to the previously reported result after high-temperature annealing. Hence, sputtered AlN NL in combination with high-temperature annealing is a promising technique for growing high-quality AlN films on sapphire substrate with a low TD density [41].

**Figure 13:**
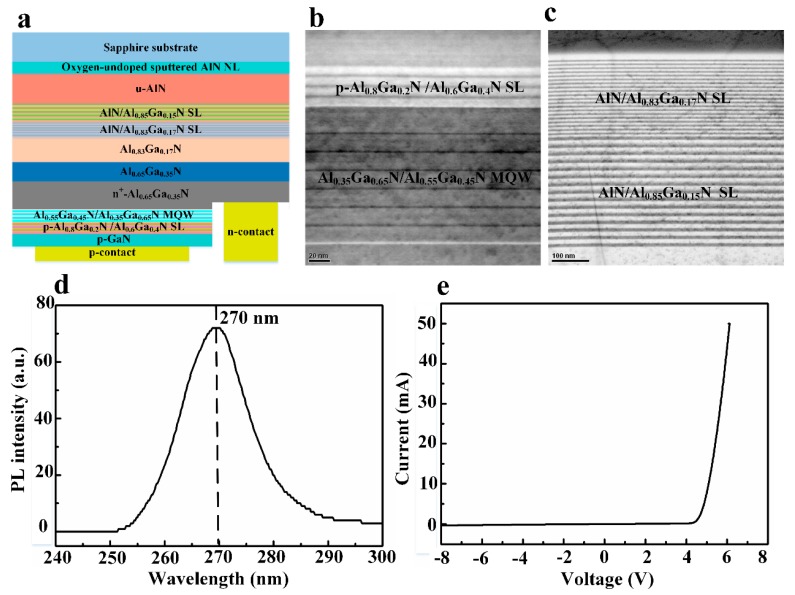
(**a**) Schematic representation of the deep-ultraviolet light-emitting diode (DUV LED) structure. (**b**,**c**) Cross-sectional transmission electron microscopy (TEM) images of the DUV LED epitaxial structure. (**d**) Photoluminescence (PL) spectrum (T = 300 K) of the DUV LED. (**e**) Current versus voltage characteristic of DUV LED.

The AlGaN-based DUV LED was grown on the crack-free AlN film on sapphire substrate with 25-nm-thick oxygen-undoped sputtered AlN NL. Figure 13a depicts the schematic of the DUV LED structure. Figure 13b shows a cross-sectional TEM image of Al_0.35_Ga_0.65_N/Al_0.55_Ga_0.45_N MQW and p-Al_0.8_Ga_0.2_N/Al_0.6_Ga_0.4_N SL. Figure 13c shows a cross-sectional TEM image of AlN/Al_0.85_Ga_0.15_N SL and AlN/Al_0.83_Ga_0.17_N SL. The AlN/Al_0.85_Ga_0.15_N SL and AlN/Al_0.83_Ga_0.17_N SL served as a strain release layer. The TEM images show that the heterointerface was abrupt, even in the very short period SL, suggesting a high-quality epitaxial growth of DUV LED structure. Figure 13d shows the PL spectrum of the DUV LED. The peak emission wavelength of DUV LED was 270 nm. The FWHM of PL spectrum of the DUV LED was 14 nm, which is close to the reported FWHM value in previous work [50]. Figure 13e shows the current versus voltage characteristic of DUV LED. At an injection current of 20 mA, the forward voltage of DUV LED was 5.4 V. 

## 4. Conclusions

In summary, we studied the effect of AlN NL (LT-AlN NL, oxygen-undoped sputtered AlN NL, and oxygen-doped sputtered AlN NL) on the optical transmittance, strain state, surface morphology, and crystal quality of AlN films on sapphire substrates. Owing to the addition of oxygen to sputtered AlN NL, the average optical transmittance of AlN film on sapphire substrate with oxygen-doped sputtered AlN NL was about 8% higher than that of AlN films on sapphire substrates with LT-AlN NL and oxygen-undoped sputtered AlN NL in the wavelength range of 200–270 nm. However, the AlN film on sapphire substrate with oxygen-undoped sputtered AlN NL had the smoothest surface morphology and lowest TD density. Moreover, the effect of nanoscale sputtered AlN NL thickness on strain state, surface morphology, and crystal quality of AlN film on sapphire substrate with sputtered AlN NL were analyzed. The AlN film on sapphire substrate with the optimum thickness of sputtered AlN NL showed weak tensile stress, a crack-free surface, and low TD density. We successfully grew a 270-nm DUV LED on the high-quality and crack-free AlN film. These findings present a simple technique for the production of high-quality AlN film on sapphire substrate with AlN NL, which has great potential for applications in high-performance DUV LED.

## References

[1] Guo W., Yang Z., Li J., Yang X., Zhang Y., Wang J., Chee K.W.A., Gao P., Ye J. (2017). Enhancing light coupling and emission efficiencies of AlGaN thin film and AlGaN/GaN multiple quantum wells with periodicity-wavelength matched nanostructure array. Nanoscale.

[2] Zhou S., Liu X., Yan H., Chen Z., Liu Y., Liu S. (2019). Highly efficient GaN-based high-power flip-chip light-emitting diodes. Opt. Express.

[3] Chen Y., Zhang Z., Jiang H., Li Z., Miao G., Song H. (2018). The optimized growth of AlN templates for back-illuminated AlGaN-based solar-blind ultraviolet photodetectors by MOCVD. J. Mater. Chem. C.

[4] SaifAddin B.K., Almogbel A., Zollner C., Foronda H.M., Alyamani A., Albadri A., Iza M., Nakamura S., DenBaars S.P., Speck J.S. (2019). Fabrication technology for high light-extraction ultraviolet thin-film flip-chip (UV TFFC) LEDs grown on SiC. Semicond. Sci. Technol..

[5] Zhao J., Ding X., Miao J., Hu J., Hui W., Zhou S. (2019). Improvement in Light Output of Ultraviolet Light-Emitting Diodes with Patterned Double-Layer ITO by Laser Direct Writing. Nanomaterials.

[6] Saifaddin B.K., Iza M., Foronda H., Almogbel A., Zollner C.J., Wu F., Alyamani A., Albadri A., Nakamura S., DenBaars S.P. (2019). Impact of roughening density on the light extraction efficiency of thin-film flip-chip ultraviolet LEDs grown on SiC. Opt. Express.

[7] Bryan Z., Bryan I., Mita S., Tweedie J., Sitar Z., Collazo R.Z. (2015). Strain dependence on polarization properties of AlGaN and AlGaN-based ultraviolet lasers grown on AlN substrates. Appl. Phys. Lett..

[8] He C., Zhao W., Wu H., Zhang S., Zhang K., He L., Liu N., Chen Z., Shen B. (2018). High-quality AlN film grown on sputtered AlN/sapphire via growth-mode modification. Cryst. Growth Des..

[9] Xiong H., Dai J.N., Hui X., Fang Y.Y., Tian W., Fu D.X., Chen C.Q., Li M., He Y. (2013). Effects of the AlN buffer layer thickness on the properties of ZnO films grown on c-sapphire substrate by pulsed laser deposition. J. Alloy. Compd..

[10] Schowalter L.J., Slack G.A., Whitlock J.B., Morgan K., Schujman S.B., Raghothamachar B., Dudley M., Evans K.R. (2003). Fabrication of native, single-crystal AlN substrates. Phys. Status Solidi C.

[11] Isobe H., Kawamura F., Kawahara M., Yoshimura M., Mori Y., Sasaki T. (2005). Synthesis of AlN Grains and Liquid-Phase-Epitaxy (LPE) Growth of AlN Films Using Sn-Ca Mixed Flux. Jpn. J. Appl. Phys..

[12] Kangawa Y., Toki R., Yayama T., Epelbaum B.M., Kakimoto K. (2011). Novel solution growth method of bulk AlN using Al and Li3N solid sources. Appl. Phys. Express.

[13] Schlesser R., Dalmau R., Sitar Z. (2002). Seeded growth of AlN bulk single crystals by sublimation. J. Cryst. Growth.

[14] Susilo N., Hagedorn S., Jaeger D., Miyake H., Zeimer U., Reich C., Neuschulz B., Sulmoni L., Guttmann M., Mehnke F. (2018). AlGaN-based deep UV LEDs grown on sputtered and high temperature annealed AlN/sapphire. Appl. Phys. Lett..

[15] Jeschke J., Martens M., Knauer A., Kueller V., Zeimer U., Netzel C., Kuhn C., Krueger F., Reich C., Wernicke T. (2015). UV-C Lasing from AlGaN multiple quantum wells on different types of AlN/sapphire templates. IEEE Photonics Technol. Lett..

[16] Padavala B., Frye C.D., Wang X., Ding Z., Chen R., Dudley M., Raghothamachar B., Lu P., Flanders B.N., Edgar J.H. (2016). Epitaxy of boron phosphide on aluminum nitride (0001)/sapphire substrate. Cryst. Growth Des..

[17] Sugahara T., Sato H., Hao M., Naoi Y., Kurai S., Tottori S., Yamashita K., Nishino K., Romano L.T., Sakai S. (1998). Direct evidence that dislocations are non-radiative recombination centers in GaN. Jpn. J. Appl. Phys..

[18] Ban K., Yamamoto J.I., Takeda K., Ide K., Iwaya M., Takeuchi T., Kamiyama S., Akasaki I., Amano H. (2011). Internal quantum efficiency of whole-composition-range AlGaN multiquantum wells. Appl. Phys. Express.

[19] Katona T.M., Craven M.D., Speck J.S., DenBaars S.P. (2004). Cathodoluminescence study of deep ultraviolet quantum wells grown on maskless laterally epitaxial overgrown AlGaN. Appl. Phys. Lett..

[20] Long H., Wu F., Zhang J., Wang S., Chen J., Zhao C., Feng Z., Xu J., Li X., Dai J. (2016). Anisotropic optical polarization dependence on internal strain in AlGaN epilayer grown on Al_x_Ga_1-x_N templates. J. Phys. D Appl. Phys..

[21] Miyake H., Nishio G., Suzuki S., Hiramatsu K., Fukuyama H., Kaur J., Kuwano N. (2016). Annealing of an AlN buffer layer in N2-CO for growth of a high-quality AlN film on sapphire. Appl. Phys. Express.

[22] Hakamata J., Kawase Y., Dong L., Iwayama S., Iwaya M., Takeuchi T., Kamiyama S., Miyake H., Akasaki I. (2018). Growth of High-Quality AlN and AlGaN Films on Sputtered AlN/Sapphire Templates via High-Temperature Annealing. Phys. Status Solidi B.

[23] Oh J.T., Moon Y.T., Kang D.S., Park C.K., Han J.W., Jung M.H., Sung Y.J., Jeong H.H., Song J.O., Seong T.Y. (2018). High efficiency ultraviolet GaN-based vertical light emitting diodes on 6-inch sapphire substrate using ex-situ sputtered AlN nucleation layer. Opt. Express.

[24] Freedsman J.J., Watanabe A., Yamaoka Y., Kubo T., Egawa T. (2016). Influence of AlN nucleation layer on vertical breakdown characteristics for GaN-on-Si. Phys. Status Solidi A.

[25] Hu H., Zhou S., Liu X., Gao Y., Gui C., Liu S. (2017). Effects of GaN/AlGaN/Sputtered AlN nucleation layers on performance of GaN-based ultraviolet light-emitting diodes. Sci. Rep..

[26] Pan L., Dong X., Li Z., Luo W., Ni J. (2018). Influence of the AlN nucleation layer on the properties of AlGaN/GaN heterostructure on Si (1 1 1) substrates. Appl. Surf. Sci..

[27] Ye H., Chen G., Wu Y. (2011). Structural and Electronic Properties of the Adsorption of Oxygen on AlN (1010) and (1120) Surfaces: A First-Principles Study. J. Phys. Chem. C.

[28] Zhou S., Xu H., Hu H., Gui C., Liu S. (2019). High quality GaN buffer layer by isoelectronic doping and its application to 365 nm InGaN/AlGaN ultraviolet light-emitting diodes. Appl. Surf. Sci..

[29] Signore M.A., Taurino A., Valerini D., Rizzo A., Farella I., Catalano M., Quaranta F., Siciliano P. (2015). Role of oxygen contaminant on the physical properties of sputtered AlN thin films. J. Alloy. Compd..

[30] Fritze S., Drechsel P., Stauss P., Rode P., Markurt T., Schulz T., Albrecht M., Blaesing J., Dadgar A., Krost A. (2012). Role of low-temperature AlGaN interlayers in thick GaN on silicon by metalorganic vapor phase epitaxy. J. Appl. Phys..

[31] Wang H., Zhou Q., Liang S., Wen R. (2019). Fabrication and Characterization of AlGaN-Based UV LEDs with a ITO/Ga_2_O_3_/Ag/Ga_2_O_3_ Transparent Conductive Electrode. Nanomaterials.

[32] Arslan E., Öztürk M.K., Özçelik S., Özbay E. (2019). Effects of the AlN nucleation layer thickness on the crystal structures of an AlN epilayer grown on the 6H-SiC substrate. Philos. Mag..

[33] Hu H., Zhou S., Wan H., Liu X., Li N., Xu H. (2019). Effect of strain relaxation on performance of InGaN/GaN green LEDs grown on 4-inch sapphire substrate with sputtered AlN nucleation layer. Sci. Rep..

[34] Pantha B.N., Dahal R., Nakarmi M.L., Nepal N., Li J., Lin J.Y., Jiang H.X., Paduano Q.S., Weyburne D. (2007). Correlation between optoelectronic and structural properties and epilayer thickness of AlN. Appl. Phys. Lett..

[35] Zhou S., Xu H., Tang B., Liu Y., Wan H., Miao J. (2019). High-power and reliable GaN-based vertical light-emitting diodes on 4-inch silicon substrate. Opt. Express.

[36] Nagata K., Makino H., Yamamoto T., Saito Y., Miki H. (2019). Origin of optical absorption in AlN with air voids. Jpn. J. Appl. Phys..

[37] Becerra D.L., Cohen D.A., Mehari S., DenBaars S.P., Nakamura S. (2019). Compensation effects of high oxygen levels in semipolar AlGaN electron blocking layers and their mitigation via growth optimization. J. Cryst. Growth.

[38] Yang S., Miyagawa R., Miyake H., Hiramatsu K., Harima H. (2011). Raman scattering spectroscopy of residual stresses in epitaxial AlN films. Appl. Phys. Express.

[39] Lughi V., Clarke D.R. (2006). Defect and stress characterization of AlN films by Raman spectroscopy. Appl. Phys. Lett..

[40] Bethoux J.M., Vennéguès P., Natali F., Feltin E., Tottereau O., Nataf G., Mierry P.D., Semond F. (2003). Growth of high quality crack-free AlGaN films on GaN templates using plastic relaxation through buried cracks. J. Appl. Phys..

[41] Uesugi K., Hayashi Y., Shojiki K., Miyake H. (2019). Reduction of threading dislocation density and suppression of cracking in sputter-deposited AlN templates annealed at high temperatures. Appl. Phys. Express.

[42] El-Tahawy M., Máthis K., Garcés G., Matsumoto T., Yamasaki M., Kawamura Y., Gubicza J. (2019). Type and density of dislocations in a plastically deformed long-period stacking ordered magnesium alloy. J. Alloy. Compd..

[43] Tang B., Miao J., Liu Y., Wan H., Li N., Zhou S., Gui C. (2019). Enhanced Light Extraction of Flip-Chip Mini-LEDs with Prism-Structured Sidewall. Nanomaterials.

[44] Zhou S., Hu H., Liu X., Liu M., Ding X., Gui C., Liu S., Guo L.J. (2017). Comparative study of GaN-based ultraviolet LEDs grown on different-sized patterned sapphire substrates with sputtered AlN nucleation layer. Jpn. J. Appl. Phys..

[45] McCullen E.F., Thakur J.S., Danylyuk Y.V., Auner G.W., Rosenberger L.W. (2008). Investigation of the occupation behavior for oxygen atoms in AlN films using Raman spectroscopy. J. Appl. Phys..

[46] Harris J.H., Youngman R.A., Teller R.G. (1990). On the nature of the oxygen-related defect in aluminum nitride. J. Mater. Res..

[47] Tanaka S., Shojiki K., Uesugi K., Hayashi Y., Miyake H. (2019). Quantitative evaluation of strain relaxation in annealed sputter-deposited AlN film. J. Cryst. Growth.

[48] Xiao S., Suzuki R., Miyake H., Harada S., Ujihara T. (2018). Improvement mechanism of sputtered AlN films by high-temperature annealing. J. Cryst. Growth.

[49] Miyake H., Lin C.H., Tokoro K., Hiramatsu K. (2016). Preparation of high-quality AlN on sapphire by high-temperature face-to-face annealing. J. Cryst. Growth.

[50] Li X., Le G.G., Bouchoule S., El G.Y., Patriarche G., Sundaram S., Disseix P., Reveret F., Leymarie J., Streque J. (2015). Structural and optical investigations of AlGaN MQWs grown on a relaxed AlGaN buffer on AlN templates for emission at 280 nm. J. Cryst. Growth.

